# Altered Structural Expression and Enzymatic Activity Parameters in Quiescent Ulcerative Colitis: Are These Potential Normalization Criteria?

**DOI:** 10.3390/ijms21051887

**Published:** 2020-03-10

**Authors:** Sebastian Kjærgaard, Morten M. B. Damm, Joan Chang, Lene B. Riis, Hanne B. Rasmussen, Rasmus Hytting-Andreasen, Susanne M. Krug, Jörg-Dieter Schulzke, Niels Bindslev, Mark Berner Hansen

**Affiliations:** 1 Digestive Disease Center, Bispebjerg Hospital, 2400 Copenhagen, Denmark; morten.da2m@gmail.com; 2 Wellcome Centre for Cell-Matrix Research, Division of Cell Matrix and Regenerative Medicine, Faculty of Biology Medicine and Health, University of Manchester, Manchester M16 8FB, UK; joan.chang@manchester.ac.uk; 3 Department of Pathology, Herlev Hospital, 2730 Copenhagen, Denmark; lene.buhl.riis@regionh.dk; 4 Department of Biomedical Sciences, Faculty of Health and Medical Sciences, University of Copenhagen, 2200 Copenhagen, Denmark; hanneb@sund.ku.dk (H.B.R.); bindslev@sund.ku.dk (N.B.); 5 Novo Nordisk Foundation Center for Basic Metabolic Research, Department of Biomedical Sciences, University of Copenhagen, 2200 Copenhagen, Denmark; rashytting@gmail.com; 6 Institute of Clinical Physiology/Nutritional Medicine, Department of Gastroenterology, Rheumatology and Infectious Diseases, Charité—Universitätsmedizin Berlin, 12203 Berlin, Germany; susanne.m.krug@charite.de (S.M.K.); joerg.schulzke@charite.de (J.-D.S.)

**Keywords:** Inflammatory bowel disease, ulcerative colitis, mucosal healing, mucosal barrier integrity, tight junction, short-circuit current, PGE_2_, COX-1, COX-2

## Abstract

Mucosal healing determined by endoscopy is currently the remission standard for ulcerative colitis (UC). However, new criteria for remission are emerging, such as histologic normalization, which appears to correlate better to the risk of relapse. Here, we study mucosal healing on a molecular and functional level in quiescent UC. We obtained endoscopic biopsies from 33 quiescent UC patients and from 17 controls. Histology was assessed using Geboes score. Protein and mRNA levels were evaluated for the tight junction proteins claudin-2, claudin-4, occludin, and tricellulin, as well as Cl^−^/HCO_3_^−^ exchanger DRA, and cyclo-oxygenase enzymes (COX-1, COX-2). The mucosal activity of COX-1 and COX-2 enzymes was assessed in modified Ussing chambers, measuring electrogenic ion transport (short-circuit current, SCC). Chronic inflammation was present in most UC patients. The protein level of claudin-4 was reduced, while mRNA-levels of claudin-2 and claudin-4 were upregulated in UC patients. Surprisingly, the mRNA level of COX-1 was downregulated, but was unaltered for COX-2. Basal ion transport was not affected, while COX-2 inhibition induced a two-fold larger decrease in SCC in UC patients. Despite being in clinical and endoscopic remission, quiescent UC patients demonstrated abnormal mucosal barrier properties at the molecular and functional level. Further exploration of mucosal molecular signature for revision of current remission standards should be considered.

## 1. Introduction

Ulcerative colitis (UC) is an idiopathic chronic inflammatory bowel disease (IBD), characterized by latent periods exacerbated by sudden relapses of activity with abdominal discomfort, increased stool frequency and rectal bleeding [1].

The primary objective of current management is to alleviate patient symptoms by achieving clinical remission. The second objective is to achieve endoscopic mucosal healing (eMH), since endoscopic remission is believed to correlate with improved long-term outcome and lower risk of relapse. The third objective is to maintain steroid-free remission [1,2].

It is difficult to predict long-term outcomes and relapse of UC with the current remission standards and available biomarkers. Therefore, better and/or complimentary dynamic, predictive and prognostic biomarkers are needed.

Several studies have demonstrated that histologic abnormalities may persist despite eMH, [3,4,5,6] and that histologic mucosal healing (hMH, deep remission) is associated with a reduced risk of relapse and prolongs steroid-free remission [5,7,8,9]. As such, adjustment of current therapy goals is in demand, since eMH is questionable as an adequate stand-alone predictive and prognostic biomarker for long-term outcome and risk of relapse.

Complete mucosal healing includes the normalization of barrier function. The mucosal barrier function depends mainly on the integrity, and related permeability, of the tight junction (TJ) complex located apically between epithelial cells. The TJ complex regulates passive paracellular passage of water and electrolytes and also acts as a barrier preventing passage of microbes and other potentially harmful molecules like lipopolysaccharides and other antigens [10]. The composition of the TJ proteins, both their expression patterns and distribution, and therefore “tightness” of the tissue, varies between organs and is influenced by various external stimuli such as pathogenic effector cytokines [10,11,12].

There is an association between UC disease activity and increased mucosal permeability, which is theorized to be an important pathogenic factor in IBD [13,14]. During active UC disease, the integrity of the mucosal barrier is compromised by an altered TJ structure. These alterations include reduced strand count and depth [15], downregulation of tightening proteins like claudin-4, occludin and tricellulin, and upregulation of channel-forming claudin-2, thereby leading to increased leakiness of mucosa [12,16,17]. Changes occur during the recurrent inflammatory response and are key hallmarks of UC.

Further, seeking to correct the level of cyclooxygenase (COX) enzyme activity seems necessary for complete mucosal healing. Eicosanoids derived from arachidonic acid, including prostaglandin E2, PGE_2_, are involved in both the promotion and resolution of acute inflammation, and ensuing healing of mucosa [18]. PGE_2_ also plays an important role in mucosal homeostasis by inducing chloride secretion, and is produced via COX-1 (constitutive) and COX-2 (inducible) enzyme activities. COX-2 enzyme activity is significantly upregulated during intestinal inflammation, thereby increasing the production of PGE_2_ [19]. As such, complete mucosal healing (cMH) consists not only of re-establishing mucosal barrier function, but also a normalization of COX-2 enzyme and its down-stream activities.

The objective of this descriptive study was to explore the concept of molecular/functional remission as a potential predictive and/or prognostic complementary biomarker of long-term outcome and risk of relapse. We hypothesized that patients with quiescent UC and mucosal healing defined by endoscopy and histology may still have disease activity at a molecular and functional level.

We examined mucosal healing at the molecular and functional level by determining mucosal barrier integrity (TJ protein complex profile and subcellular localization), COX-enzyme activity and ion transport capacity (basal and stimulated) as compared to clinical, endoscopic and histologic findings in UC patients with quiescent disease.

## 2. Results

**Figure 1:**
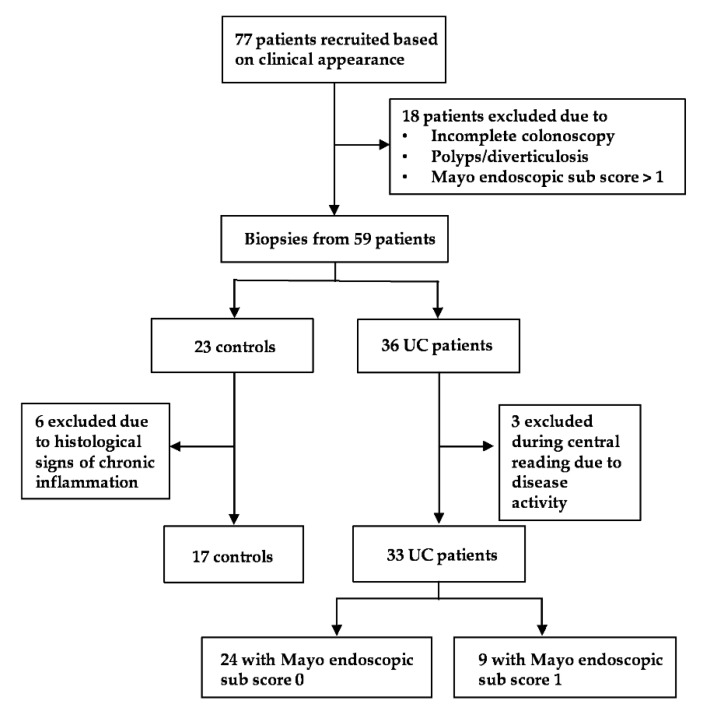
Patient recruitment flowchart.

Seventy-seven individuals were initially included based on clinical appearance. Hereof, 18 (23% 18/77) were excluded during the endoscopic procedure due to colonic polyps, diverticulosis, disease activity (Mayo endoscopic sub score > 1), or incomplete endoscopy. Three UC patients (8%, 3/36) were excluded during the central reading process of endoscopy due to disease activity. Six controls (26%, 6/23) were excluded after histological examination due to signs of subclinical chronic inflammation. As a result, we enrolled 33 quiescent UC patients in clinical and endoscopic remission and 17 endoscopically and histologically healthy controls (Figure 1). There were no apparent important differences between groups with respect to gender, age, smoking habit or use of medication except for the use of 5-ASA and other immune modulating medications in UC patients (Table 1).

### 2.1. Clinical and Endoscopic Assessment

All 33 UC patients were in clinical and endoscopic remission and had normal stool frequency. The majority (82%, 27/33) had been in clinical remission for more than 3 months prior to study inclusion. Most UC patients (73%, 24/33) had a Mayo endoscopic sub score of 0, and only a minority (27%, 9/33) had a Mayo endoscopic sub score of 1.

### 2.2. Histological Assessment

**Figure 2:**
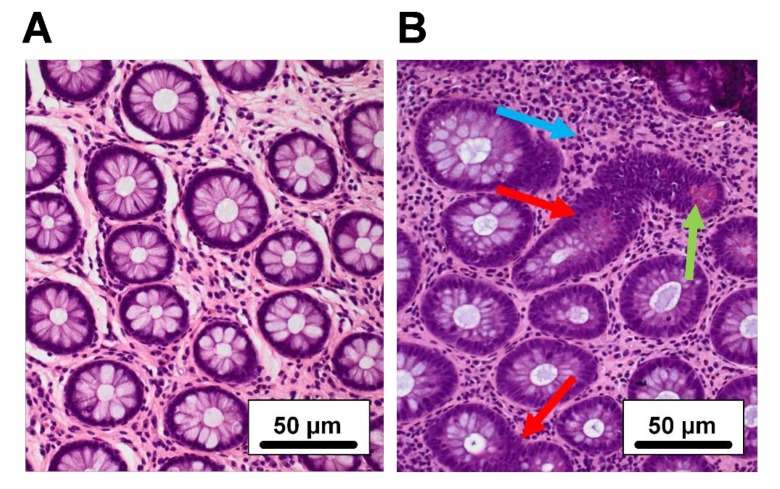
Histological features in quiescent ulcerative colitis (UC). H&E staining of colonic mucosal biopsies (×200 magnification). Two UC patients, both in clinical and endoscopic remission but with different histologies: (**A**) No inflammation, (**B**) chronic inflammation. *Blue arrow:* increased chronic inflammatory infiltrate with lymphocytes. *Green arrow:* Paneth cell metaplasia. *Red arrows:* crypt irregularity.

Biopsies from all patients were examined histologically and scored according to the Geboes score. Regardless of the Mayo endoscopic sub score, the majority (66%, 22/33) of the UC patients had signs of mild to moderate chronic inflammation (defined by increased lymphocyte infiltration and Geboes score 1.1-1.2), while the remaining (33%, 11/33) patients showed no signs of inflammation (Figure 2). No acute inflammation, defined primarily as the presence of neutrophils, was observed in any of the biopsies. All controls presented normal histology.

### 2.3. Mucosal Integrity Assessment

#### 2.3.1. Protein levels

**Figure 3:**
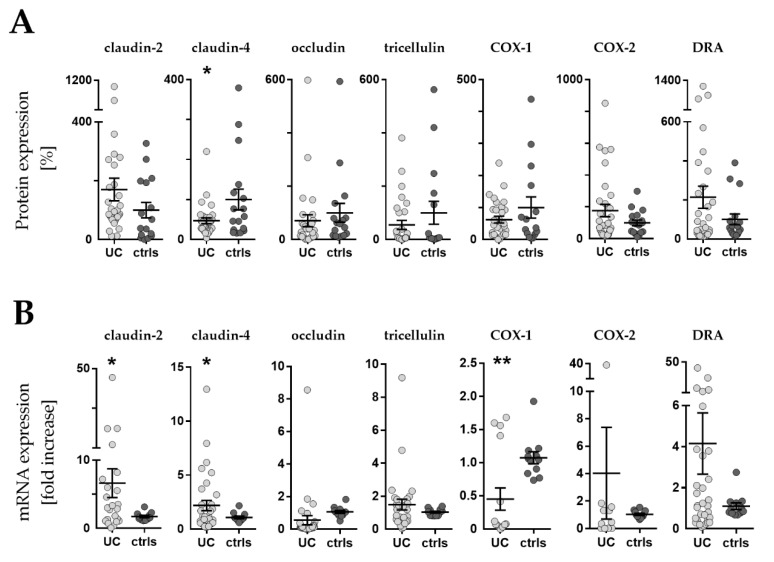
Western blot and RT-qPCR. (**A**) Expression levels of barrier related proteins by densitometric analysis of Western blot (WB). Values are expressed as % of a mean of all controls (ctrls). Bars indicate mean ± standard error of the mean. Asterisk indicates statistically significant difference between groups (* *p* < 0.05). (**B**) mRNA levels by RT-qPCR. Values are expressed relative to a mean of all controls. Three controls (18%, 3/17) presented a different baseline of housekeeping genes, while two controls (12%, 2/17) presented outliers, which were removed, leaving the final number of controls for mRNA analysis at 12 (70% 12/17). One outlier (3%, 1/33) was removed from the UC group. *n* = number of UC observations: COX-1 (*n* = 16), COX-2 (*n* = 11), claudin-2 (*n* = 23), claudin-4 (*n* = 33), occludin (*n* = 31), tricellulin (*n* = 29), and DRA (*n* = 33). Bars indicate mean ± standard error of the mean. Asterisks indicate statistically significant difference between two groups (* *p* < 0.05, ** *p* < 0.01).

Figure 3A shows the protein expression of tight junction proteins claudin-2, claudin-4, occludin, and tricellulin, as well as Cl^−^/HCO_3_^−^ exchanger downregulated-in-adenoma (DRA), COX-1 and COX-2 enzymes. UC patients demonstrated a 55% reduced expression of claudin-4 protein level compared to controls (*P* = 0.035), while levels of TJ proteins claudin-2, occludin, and tricellulin were unaltered. Protein levels of COX-1, COX-2 and DRA were also unaltered.

#### 2.3.2. mRNA levels

Figure 3B shows mRNA expression of the same proteins, where detection was possible. In UC patients, mRNA levels were significantly upregulated for both claudin-2 (5-fold, *P* = 0.030) and claudin-4 (2-fold, *P* = 0.031). Occludin, tricellulin and DRA mRNA were unaltered. COX-1 mRNA levels were significantly decreased (2-fold, *P* = 0.003), while COX-2 was unaltered in UC patients.

#### 2.3.3. Protein Localization by Fluorescent Immunohistochemistry

We next examined the cellular and subcellular localization of Cl^−^/HCO_3_^−^-exchanger DRA as well as TJ proteins occludin and claudin-4 in colonic biopsies from 11 UC patients and 11 controls using fluorescent immunohistochemistry. Localization of claudin-2 in controls was attempted using four different antibodies without achieving specific staining (see Materials and Methods).

**Figure 4:**
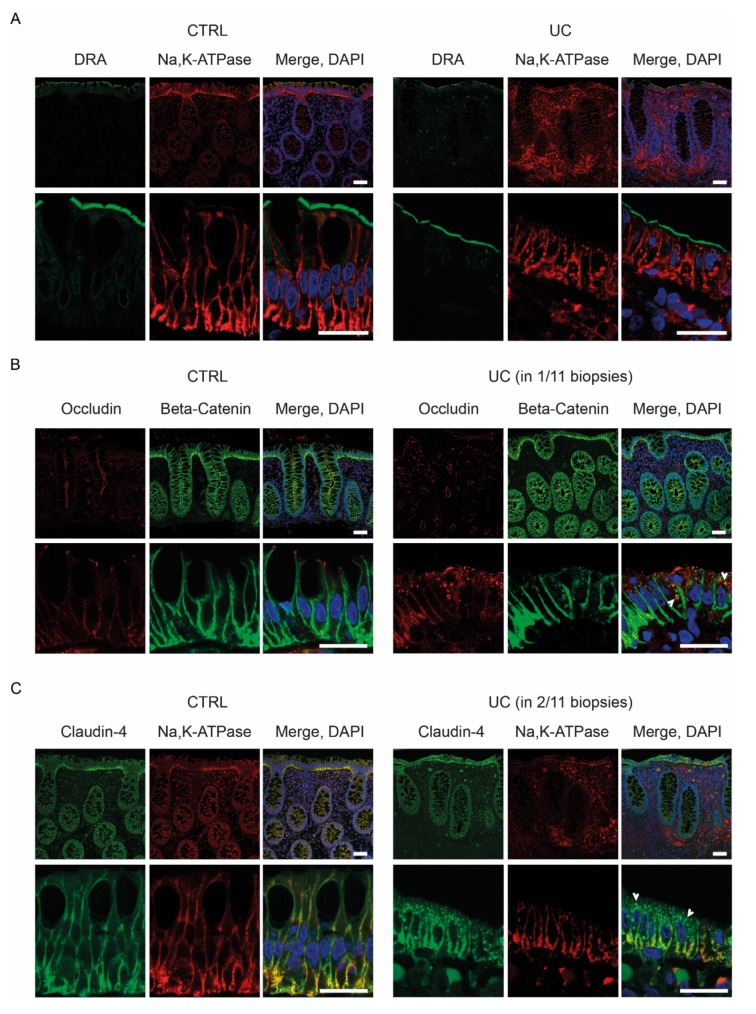
The localization of downregulated-in-adenoma (DRA), occludin and claudin-4. Representative confocal images of human colonic biopsies from control, CTRL, (*n* = 11) and UC patients (*n* = 11) stained for (**A**) DRA, (**B**) occludin and (**C**) claudin-4. Occludin and claudin-4 display intracellular accumulation in the surface epithelium of a subset of UC patients (white arrows). Stains for Na^+^/K^+^-ATPase or beta-catenin were included to mark the lateral membranes and the nuclei were visualized with DAPI. The localization depicted for UC patients was observed in the indicated subset of biopsies (2/11, 1/11, respectively). The upper panels in **A**–**C** show low magnification images and below high magnification images, while the lower panels show high magnification images of the surface epithelium. Scale bars: upper panel in **A**–**C**: 50 µm, lower panels in **A**–**C**: 20 µm.

In controls, DRA was localized to the apical microvilli of the surface epithelium (Figure 4A). In addition to the strong apical localization, a weak intracellular signal was observed in the surface cells, concentrated around the nucleus and most likely originating from the endoplasmic reticulum. DRA was noticeably absent from goblet cells. Occludin was detected at the TJ of both surface and crypt cells, with the strongest expression observed in crypts (Figure 4B). Claudin-4 was detected at lateral membranes as well as the TJ. The expression was mainly detected in the surface epithelium; however, a weaker signal was also observed in crypt cells (Figure 4C). In addition to the membrane localization at lateral membranes and TJ, claudin-4 was occasionally observed in intracellular vesicles in the surface epithelium (Figure 4C).

For the major part of analyzed UC biopsies (81%, 9/11 biopsies), no significant changes in the localization of DRA, occludin and claudin-4 were observed (data not shown). However, for the remaining two UC biopsies (19%, 2/11), significant alterations in the localization of primarily claudin-4, but also occludin, were observed. The two UC biopsies were characterized by surface epithelial cells of low height that displayed substantial accumulation of claudin-4 in intracellular vesicles (Figure 4C). Similarly, but less significant, accumulation was observed for occludin in one of the two biopsies (Figure 4B). Interestingly, Na^+^/K^+^-ATPase, and to a lesser extent beta-catenin, demonstrated some vesicular accumulation in the surface epithelium, although both were included as markers of lateral membranes (Figure 4C). The localization of DRA was not significantly disturbed (Figure 4A).

### 2.4. Transport Characteristics

**Figure 5:**
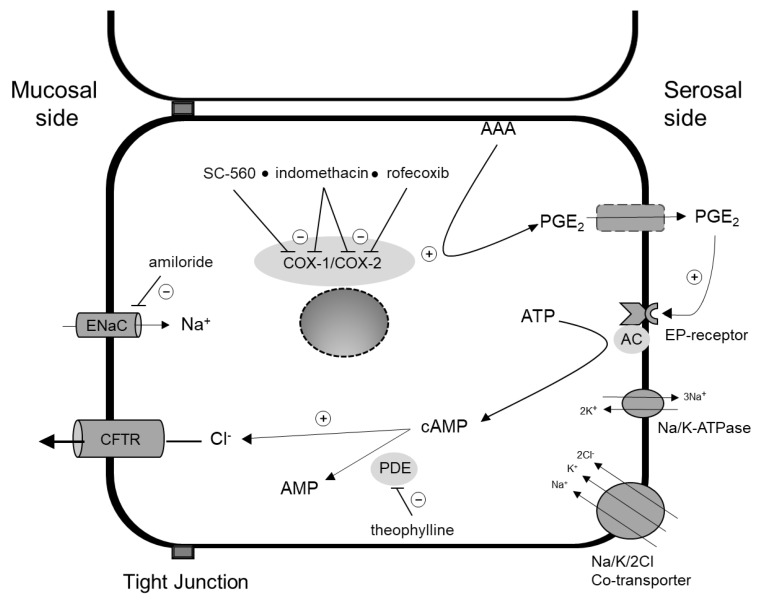
Prostaglandin E2 (PGE_2_)-dependent chloride (Cl^−^) secretion in a colonocyte. Cyclooxygenase enzyme 1 (COX-1) and -2 (COX-2) produce PGE_2_ from activated arachidonic acid (AAA). PGE_2_ leaves the cell through the basolateral membrane and exerts its function either by auto- or paracrine stimulation. Binding to its receptor (EP-receptor) stimulates the synthesis of second messenger cyclic adenosine monophosphate (cAMP) from adenosine triphosphate (ATP) by adenylate cyclase (AC). cAMP in turn stimulates luminal Cl^−^ secretion and is degraded to adenosine monophosphate (AMP) by phosphodiesterase-enzymes (PDE). SC-560 and rofecoxib specifically inhibits COX-1 and COX-2, while indomethacin is a non-specific COX-inhibitor. Theophylline is a non-specific phosphodiesterase inhibitor. Amiloride inhibits Epithelial Sodium Channel (ENaC)-mediated luminal sodium absorption. Chloride influx through basolateral Na^+^/K^+^/2Cl^−^-cotransporter and efflux through apical chloride channel cystic fibrosis transmembrane conductance regulator (CFTR). PGE_2_ synthesis also occurs in the subepithelium. + and – indicate stimulatory and inhibitory effects, respectively.

**Figure 6:**
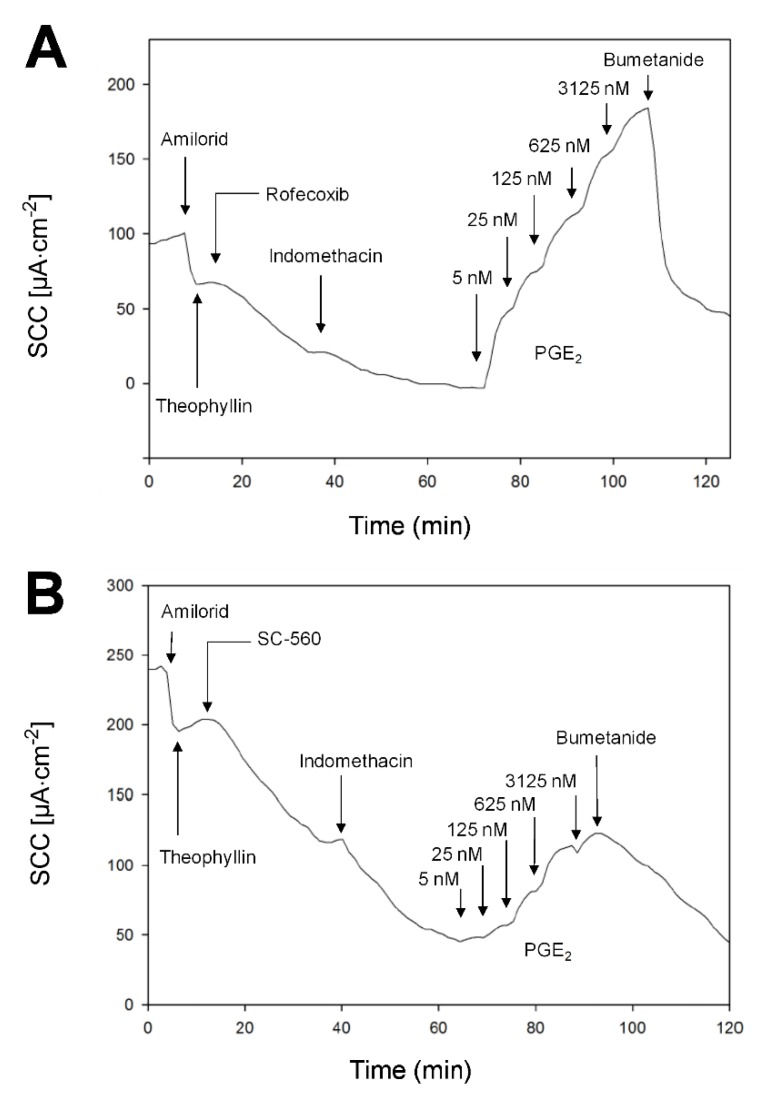
Examples of short-circuit current (SCC) data. Measurements in biopsies from an UC patient (**A**), and control (**B**). Biopsies mounted in the mini-Ussing-air-suction (MUAS) chambers were exposed to: amiloride (sodium absorption inhibitor, 20 µM), theophylline (non-specific phosphodiesterase inhibitor, 400 µM), a specific COX inhibitor (either COX-1, SC-560, or COX-2, rofecoxib, 500 nM) followed by indomethacin (non-specific COX inhibitor, 13 µM), prostaglandin E2 (PGE_2_) in increasing concentrations (five-step, factor five from 5 to 3125 nM), and ultimately either bumetanide (Na^+^/K^+^/2Cl^−^-cotransporter inhibitor, 25 µM) or ouabain (Na^+^/K^+^-ATPase inhibitor, 200 µM).

Figure 5 illustrates the PGE_2_–induced secretion pathway, while Figure 6 shows two examples of mini-Ussing-air-suction (MUAS-chamber) experiments illustrating short-circuit current (SCC)-responses to pharmacological intervention targeting specific components of the secretion pathway. The results are listed in Table 2.

**Figure 7:**
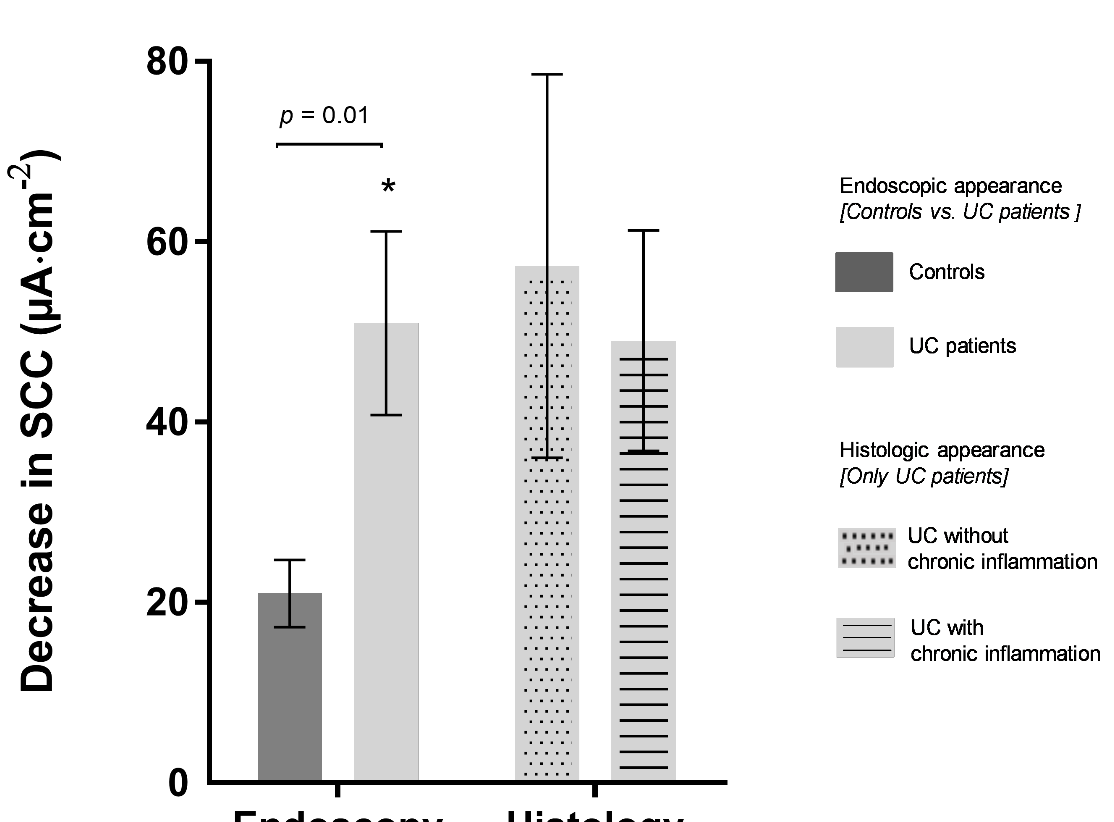
Mucosal function, expressed as a decrease in short-circuit current (SCC) after COX-2 enzyme inhibition with rofecoxib, correlated with endoscopy and histology for patient groups. Endoscopy (left): comparing functional data for controls (*n* = 11) to UC patients in clinical and endoscopic remission (*n* = 28). Histology (right): comparing functional data for UC patients with (*n* = 19) and without (*n* = 9) chronic inflammation. A larger decrease in SCC indicates elevated levels of PGE_2_. Values are expressed as mean ± SEM. Asterisk indicates statistically significant difference between two groups (* *p* = 0.01).

On average, UC patients demonstrated a 2.5-fold larger decrease in SCC to COX-2 inhibition with rofecoxib (*P* = 0.01), indicating an increased COX-2 activity and elevated levels of PGE_2_ in UC patients (Figure 7). Ten UC patients (35%, 10/28) demonstrated a COX-2 activity distinctly higher than in controls, defined as control mean + 2 SD. Half of these (5/10) were UC patients with a Mayo endoscopic sub score of 0, while the remaining half had a Mayo endoscopic sub score of 1. The decrease in SCC following COX-2 inhibition was similar in UC patients regardless of their status in histology-proven chronic inflammation (Figure 7).

We found no differences in baseline SCC between UC patients and controls, even after inhibiting ENaC-mediated sodium absorption with amiloride, indicating a normal overall ion transport for quiescent UC patients. Nor did we detect any differences in SCC increase in response to the non-specific PDE-inhibitor theophylline between UC patients and controls, indicating equal basic cyclic-nucleotide monophosphate activity.

Following the inhibition of both COX-1 and COX-2 enzymes, thereby eliminating local endogenous tissue-production of PGE_2_, we added exogenous PGE_2_ in cumulated concentration steps by a factor of five in five steps (5-3125 nM) (Figure 6). A double Michaelis–Menten equation provided the best fit for the observed increases in SCC, indicating a contribution to SCC increase most likely from two different receptors; a high- and a low-affinity subtype. The high-affinity receptor responded to concentrations down to a few nM with a half maximal effective concentration (EC_50_) of 11.0 ± 2.4 nM, while EC_50_ of the low-affinity receptor was 493 ± 111 nM. There was no significant difference between UC patients and controls in terms of EC_50_ and maximum response to PGE_2_ (R_max_) (Table 2).

## 3. Discussion

The present study provides data suggesting that some patients with quiescent UC disease, in clinical and endoscopic remission, still maintain signs of disease activity at the molecular and functional level. With that aspect, we propose to consider and further explore complementing endoscopic and histologic assessment with molecular (structural TJ proteins) and functional (COX enzyme activity) markers, in order to define complete mucosal healing (cMH), which would be the ultimate biomarker index and target-to-treat for mucosal healing and predicting long-term outcomes and risk of relapse.

### 3.1. Mucosal Barrier Function

We demonstrate that in quiescent UC, claudin-4 protein level is downregulated, despite upregulation at the mRNA level, indicating a potential accelerated degradation of the protein. In support of this, a subset of UC patients displayed displacement of claudin-4 from the cell surface to intracellular vesicles (Figure 4C).

The mRNA expression of claudin-2 was upregulated with unchanged protein levels, indicating activity on a molecular level, albeit not detectable using Western blot (WB). Our findings are in line with Vivinus et al., who demonstrated that the expression of other important TJ proteins (occludin, ZO-1 and alpha-catenin) was reduced at the level of mRNA in quiescent IBD. The protein levels of TJ proteins were, however, not measured in their study [20].

A compromised barrier in quiescent UC is likely associated with ongoing clinical symptoms [20,21,22]. Chang et al. reported that 17% of UC patients suffered from diarrhea and/or abdominal pain despite endoscopic mucosal healing. They demonstrated an association between symptoms and increased intestinal permeability evaluated by endoscopic confocal laser endomicroscopy, which is a relatively novel endoscopic tool measuring fluorescein leakage in vivo, an indirect marker of intestinal barrier integrity and function [21]. In another study, in patients with Crohn’s disease (CD) in endoscopic remission, fluorescein leakage proved to be a useful predictor for relapse of CD [23]. So far, no similar follow up has been reported for UC patients, although Karstensen et al. did observe fluorescein leakage in 82% of patients with active UC [24]. As such, the data support that an intact mucosal barrier function is likely to be a reasonable target-to-treat, and that endoscopic confocal laser endomicroscopy has the potential of being part of the assessment toolbox, together with endoscopy and molecular signatures, of MH, disease activity and response to therapy.

### 3.2. Ion Transport and COX-Enzyme Activity

Besides barrier function, we studied the mucosal ion transport properties. In active disease, epithelial ion transport is impaired in terms of the reduced absorption of sodium through epithelial sodium channels, ENaC, and chloride through the Cl^−^/HCO_3_^−^ exchanger, DRA, while secretion remains unaltered [25,26]. In agreement with Gustafsson et al., we found no difference in basal ion transport between patients with quiescent UC vs. controls [27]. Following in vitro drug intervention, we studied different components of the PGE_2_-dependent chloride secretion signaling pathway, including, indirectly, the enzyme activities of COX-1 and COX-2.

Our MUAS experiments on biopsies from a large subset of UC patients in clinical and endoscopic remission revealed a greater response towards COX-2 inhibition compared to the colon-healthy controls, indicating increased COX-2 activity as well as elevated levels of PGE_2_. This corresponds well with our knowledge of COX-2 being upregulated during active disease and resulting in an increased production of PGE_2_ [28,29]. However, the altered epithelial COX-2 activity appears not to be dependent on the mild to moderate chronic inflammation observed in most UC patients. This indicates basic alterations in UC patients compared to controls and challenges histology as a stand-alone marker for mucosal integrity at a deeper level (Figure 7).

Unexpectedly, we found significantly reduced levels of COX-1 mRNA, which might be due to a decreased population of COX-1-expressing intestinal tuft cells in quiescent UC patients (unpublished data), although COX-1 protein levels were unaltered. The fact that both the qPCR and WB results include subepithelial expressions might explain why the results do not reflect epithelial function, as measured in Ussing chambers (Table 2).

It is important to gain further information on PGE_2_ receptors, as they mediate PGE_2_ effects in acute inflammation and are associated with the development of colorectal cancer as well as other serious complications related to IBD [18,30]. The application of exogenous PGE_2_ provided data indicating the presence of two distinct functional PGE_2_ receptors, a high- and a low-affinity receptor. Of potential importance, the high-affinity receptor was sensitive to concentrations of nM PGE_2_ (EC_50_ of about 11 nM). We speculated that UC patients have increased sensitivity and higher potency at lower levels of PGE_2_ concentrations and thereby increased activity of the high-sensitive PGE_2_ receptor. However, this was not the case in the present small cohort, as high-affinity EC_50_s were not different between patient groups (Table 2). With the existence of four PGE_2_ receptor subtypes, more experiments are required to relate the two observed receptor subtypes to either of the four receptors.

The implementation of Ussing chamber technique in assessing disease activity alongside endoscopic and histologic parameters is impractical, but our results illustrate the necessity of reviewing the current remission standard including molecular and functional markers.

### 3.3. Limitations of Study

Firstly, the strict patient selection resulted in the notorious inherent slow recruitment process. Since the final number of included UC patients was low, we had to pool all into one group regardless of the Mayo endoscopic sub scores. Secondly, several UC patients were referred for only a sigmoidoscopy, which would not detect polyps or other uncharacteristic non-continuous activity in the right and transverse segments of the colon. Thirdly, we find no apparent differences in basic characteristics between groups but, considering the complexity of UC and the many influencing factors, these results should be confirmed on a larger number of patients. Further, we did not include any information on the content of dietary consumption and the use of probiotics, which are both important factors in mucosal healing. Evaluation of such parameters is clearly desirable for future studies on UC.

### 3.4. Perspectives

The ultimate therapeutic goal is the long-term complete remission of patient signs and symptoms as well as disease activity biomarkers. For UC, improved mucosal healing (MH) is of particular importance, as it correlates reversely with risk of progression, developing serious complications and relapse of disease [31]. However, the definition of MH is being questioned and additional predictive and prognostic biomarkers are clearly needed.

MH is currently assessed by endoscopy and it is debated whether the assessment of histology should also be included for disease predictions [8,32,33]. A patient is in “deep remission” if both endoscopy and histology show normal findings. Whether deep remission is sufficient as a treatment target or if normalization of additional mucosal functions is needed to improve clinical outcomes and the course of the disease inclusive of the time to relapse remain open questions.

This study explores potential means of how to define and assess mucosal healing at the molecular and functional levels. Indeed, it seems appropriate to consider and further explore such biomarkers as treatment-to-target concepts. From the results of the present study, tight junction protein claudin-4 expression is a worthwhile readout parameter in particular, eventually being related to claudin-2 expression.

## 4. Materials and Methods

### 4.1. Study Population

UC patients were all in clinical and endoscopic remission, i.e., total Mayo score ≤ 2, and no sub score > 1, and undergoing routine sigmoid or colonoscopy examination for disease control and monitoring. The control group consisted of patients referred to colonoscopy on suspicion of colorectal disease but deemed healthy based on results from endoscopic and histologic examinations. Patients were excluded from the study if regularly treated with a non-steroidal anti-inflammatory drug (NSAID), non-selective COX-inhibitor, and/or suffered from other chronic gastrointestinal (GI) diseases; i.e., dyspepsia, celiac disease, lactose intolerance, irritable bowel syndrome, diverticulosis, and/or neoplasia coli.

We enrolled 33 UC patients and 17 controls. Four UC patients (12%, 4/33) and eight controls (47%, 8/17) suffered from co-morbidities including hypercholesterolemia, hypothyroidism, asthma, epilepsy, primary sclerosing cholangitis, nephrectomy, fibromyalgia, depression, and hypertension. The study population characteristics and medication are listed in Table 1.

### 4.2. Biopsy Procedure

Seven sigmoid biopsies were obtained from each patient during endoscopy, approximately 30 cm proximal to the anal verge on retraction of the endoscope using standard biopsy forceps (Boston Scientific, Radial Jaw 4, outside diameter of 2.2 mm). Four biopsies were immediately transferred to an iced bicarbonate Ringer solution with the following composition (in mM): Na^+^ (140), K^+^ (3.8), Cl^−^ (117), Ca^2+^ (1.0), Mg^2+^ (0.5), SO_4_^2−^ (0.5), HCO_3_^−^ (25), and D-glucose (5.5) to be analyzed in MUAS chambers [34]. Two biopsies were snap-frozen in liquid nitrogen and stored at −80 °C for the further quantification of protein and mRNA expression. One biopsy was preserved in paraformaldehyde for histological and immunohistochemical examination.

### 4.3. Chemicals, Antibodies and Primers

All chemicals for the MUAS experiments were purchased from Sigma-Aldrich (Seelze, Germany). Western blot antibodies for claudin-2 (cat. no.: 51-6100), claudin-4 (cat. no.: 329400), and occludin (cat. no.: 71-1500) were purchased from Invitrogen (Karlsruhe, Germany); tricellulin (cat. no.: 700191) from Abfinity Thermofisher Science (Karlsruhe, Germany); COX-2 (cat. no.: M3210) from Spring Biosciences; COX-1 (cat. no.: ABIN343669), SLC26A (DRA; cat. no.: ABIN2777377), and GAPDH (cat. no.: ABIN3187999) were purchased from Antibodies-online.com. Immunofluorescence antibodies for claudin-4 (cat. no.: 32-9400) were purchased from Thermo Fisher Scientific (Roskilde, Denmark); occludin (cat. no.: SC-133265), SLC26A (DRA; cat. no.: SC376187), Beta-catenin (cat. no.: SC-7963), and Na^+^/K^+^-ATPase (cat. no.: SC-28800) were purchased from Santa Cruz Biotechnology (Heidelberg, Germany). Primers for quantitative reverse transcription-polymerase chain reaction (qRT-PCR) were ordered from PrimerDesign (Camberley, UK) or Eurofins MWG (Ebersberg, Germany). The primer sequences used were: COX-1 (*PTGS1*) forward (5′- TTGGGGAGAGTATGATAGAGATTG -3′) reverse (5′- CGGAAGGAAACGTAGGGACAG -3′); COX-2 (*PTGS2*) forward (5′- CAGGCTTCCATTGACCAGAG -3′) reverse (5′- TTTCTCCTGTAAGTTCTTCAAATGAT -3′); Claudin-2 (*CLDN2*) forward (5′- ATCAGTGCCCCATTTGTACC -3′) reverse (5′- TCTCTCTGCCAGGCTGACTT -3′); Claudin-4 (*CLDN4*) forward (5′- CGCACAGACAAGCCTTACTC -3′) reverse (5′- CTCAGTCCAGGGAAGAACAAAG -3′); Occludin (*OCLN*) forward (5′- AGCAGCGGTGGTAACTTTG -3′) reverse (5′- AGTTGTGTAGTCTGTCTCATAGTG -3′); Tricellulin (*MARVELD2*) forward (5′- GGACAGATAGCTGCAATGATCTTC -3′) reverse (5′- GCTCATTTATCTCCTGTTGTTCCATA -3′); DRA (*SLC26A3*) forward (5′- CCAGATCAGCAGTTCAGGAGAG -3′) reverse (5′- CCAGGAGAAATCCAATGGCTAGA -3′); GAPDH forward (5′- GAGTCAACGGATTTGGTCGT -3′) reverse (5′- GACAAGCTTCCCGTTCTCAG -3′).

### 4.4. Study Methods

Six complementing methods for assessing mucosal status were employed, (A) clinical assessment and endoscopy, (B) histology, (C) constituent protein levels evaluated by 3 methods: (C.1) protein expression by WB and densitometric analysis, (C.2) mRNA expression by qRT-PCR, (C.3) localization of selected proteins by immunofluorescence, and finally (D) transport capabilities by MUAS technique [34].

#### 4.4.1. Clinical Assessment and Endoscopy

Clinical symptoms were scored according to the Mayo score before the endoscopic procedure. Endoscopies were performed and assessed by local physicians and were filmed and assessed externally according to the Mayo endoscopic sub score (central reading). Any discrepancy between local and external readings would turn out in favor for the latter.

#### 4.4.2. Histology

Biopsies were fixed immediately in paraformaldehyde and then embedded in paraffin. Sections, 4 µm thick, were stained with hematoxylin and eosin (H&E). Each biopsy was assessed for inflammatory activity and scored blindly by a GI expert pathologist according to the Geboes score [3]. Acute inflammation was defined as the presence of neutrophil infiltration, and chronic inflammation by an increased lymphocyte infiltration.

#### 4.4.3. Mucosal Proteins and Function

##### Western Blot

Tissue, suspended in a lysis buffer, 20 mM TRIS, 5 mM MgCl_2_, 1 mM EDTA, 0.3 mM EGTA, containing protease inhibitors (cOmplete, Roche, Basel, Switzerland) was homogenized using a FastPrep24 Homogenizer (MP Biomedicals, Eschwege, Germany), followed by centrifugation at 200× *g* for 5 min (at 4 °C) and a subsequent centrifugation of the remaining supernatant at 43,000× *g* for 30 min (at 4 °C). The resulting pellets contained the membrane proteins and were dissolved in resuspension buffer, 10 mM TRIS-Cl pH 7.5; 150 mM NaCl, 0.5% Triton X-100, 0.1% SDS, and protease inhibitors. Protein concentrations were determined using BCA Protein assay reagent, Pierce (Perbio Science, Bonn, Germany), quantified with a plate reader, (Tecan, Crailsheim, Germany). Protein samples of the same concentrations were prepared and mixed with Laemmli buffer, denatured at 95 °C for 5 min, separated on SDS polyacrylamide gels, and transferred to PVDF membranes, Perkin Elmer (Rodgau, Germany).

Proteins were detected by immunoblotting with primary antibodies against claudin-2 and claudin-4, occludin, tricellulin, COX-1, COX-2, SLC26A (DRA) and GAPDH. After washing steps in TBS/0.1% Triton X-100, TBST, membranes were incubated with secondary anti-mouse or anti-rabbit antibodies. Membranes were washed and incubated with Lumilight, Roche, and specific signals were detected with a chemiluminescence imager, Fusion FX7, Vilber, and analyzed using a quantification software, AIDA, Raytest (Straubenhardt, Germany).

##### mRNA Levels by Quantitative Real-time PCR

RNA was extracted using the RNeasy Mini Kit, Qiagen (Copenhagen, Denmark), following the manufacturer’s protocol with changes in tissue disruption: up to 30 mg of tissue was placed in a 2 ml Eppendorf tube together with a metal bead and 350 µL RLT/0.01% *v/v* 2-mercaptoethanol. The tubes were placed in a Tissue Lyser, Qiagen (Copenhagen, Denmark), for 3 × 2 min at 50 Hz, and samples were centrifuged at 8,000× *g* for 3 min. The supernatant was used for the extraction.

Template cDNA was generated from 500 ng of total extracted RNA using TaqMan^TM^ Reverse Transcription kit, Applied Biosystems (Warrington, UK), according to manufacturer’s instructions in the presence of RNaseOUT. The following conditions were used for qRT-PCR in a CFX384 Touch^TM^ Real-Time PCR Detection System, Biorad (Watford, UK): 95 °C for 10 min, followed by 45 cycles of 95 °C for 15 s and 58 °C for 1 min; this was proceeded with 95 °C for 15 s, 60 °C for 15 s, and finally 95 °C for 15 s.

##### Protein Localization by Fluorescent Immunohistochemistry

Twenty-two biopsies, 11 UC and 11 controls, were fixed in paraformaldehyde, embedded in paraffin and cut into 4 µm thick sections. The sections were deparaffinized and rehydrated in a xylene–ethanol series and subjected to antigen retrieval by boiling in 1 mM EDTA pH 8.0 for a total of 10 min. Unspecific binding was blocked for 30 min in PBS containing 0.1% Triton X-100 and 0.2% fish skin gelatin. Primary antibodies diluted in the same buffer were added overnight at 4 °C. The following antibodies were employed (dilution in parenthesis): mouse anti-occludin (1:100), mouse anti-SLC26A3 (1:100), mouse anti-beta-Catenin (1:100), rabbit anti-Na^+^,K^+^-ATPase (1:100), and mouse anti-Claudin-4 (1:100). Four antibodies directed against Claudin-2 were tested on control biopsies without obtaining a specific signal (#32-5600 and #51-6100 from Thermo Fischer Scientific, Roskilde, Denmark, #28530 from Cell Signaling Technology, BioNordika, Herlev, Denmark, and sc-293233 from Santa Cruz Biotechnology, Heidelberg, Germany). Bound primary antibodies were detected by incubation in AlexaFluor®-conjugated secondary antibodies, Thermo Fisher Scientific (Roskilde, Denmark), for 1 hour at room temperature. Nuclei were detected using 4′6-diamidino-2-phenylindole (DAPI), Thermo Fisher Scientific (Roskilde, Denmark). Sections were mounted in Prolong Diamond, Thermo Fisher Scientific (Roskilde, Denmark). Confocal images were acquired with Zeiss LSM 710/LSM780 confocal microscopes using ×20 objective, NA 0.8, or ×63 oil immersion objective, NA 1.4. Pinhole size was 1 airy unit, the pixel format 1024 × 1024 and line averaging was employed to reduce noise.

#### 4.4.4. Mini-Ussing-Air-Suction (MUAS) Chamber Technique

Within 45 min after collection, the biopsies were mounted in MUAS-chambers as previously described [34]. Both the serosal and mucosal side were bathed in bicarbonate-Ringer solution. Baseline electrogenic mucosal ion transport properties (expressed as short-circuit-current, SCC) before pharmacological intervention were measured approximately 10 min after mounting. Biopsies were exposed to pharmacological intervention with amiloride (epithelial sodium channel, ENaC, inhibitor, 20 µM), theophylline (non-specific phosphodiesterase, PDE, inhibitor, 400 µM), indomethacin (non-selective COX inhibitor, 13 µM), SC-560 (selective COX-1 inhibitor, 500 nM), rofecoxib (selective COX-2 inhibitor, 500 nM), and to PGE_2_ in increasing concentrations (5-step, factor 5 from 5 to 3125 nM), exploring various components of the PGE_2_–induced secretion pathway. All biopsies were exposed to bumetanide (Na^+^/K^+^/2Cl^−^-cotransporter inhibitor, 25 µM) and/or ouabain (Na^+^/K^+^-ATPase inhibitor, 0.2 mM) at the end of the experiment to verify the viability of the biopsies, judged as an adequate response due to former manipulations. Amiloride was added to the mucosal side of the biopsies mounted in MUAS-chambers, while all other compounds, including bumetanide and ouabain, were added to the serosal side. Luminal chloride secretion was measured as SCC after inhibiting electrogenic luminal sodium absorption. As such, SCC is a result of the combined activity of enzymes involved in the signaling cascade. By manipulating a specific component at a time in the pathway, the concomitant change in SCC was assumed as an indirect measurement for a change in the related enzyme activity.

### 4.5. Statistical Analysis

Values are expressed as mean ± SEM. Mean values were used if identical experiments on biopsies from the same patient were performed. Student’s *t*-test with Welch’s correction was used to identify differences between two groups, except for densitometric analyses of WB, where we used the *t*-test with Bonferroni correction. A *p*-value < 0.05 was considered significant.

## 5. Conclusions

Most UC patients in the present study demonstrated histological signs of chronic inflammation and compromised mucosal barrier functions during quiescent disease. The mucosal barrier and function signatures included altered TJ protein barrier composition, increases in COX-2 enzyme activity and related ion transport properties.

Moving forward, we propose to consider and continue evaluating mucosal healing using molecular and functional markers as potential target-to-treat endpoints, in addition to endoscopic and histological markers, for UC remission. Our results suggest that particularly assessing claudin-4 expression could prove useful in this respect.

## Figures and Tables

**Table 1:** Study population characteristics.

	UC	Controls
Total number	33	17
Males/females	15/18	8/9
Age, mean, years (range)	39 (23–75)	46 (20–68)
Smoking habit, active/ex-smoker/never	2/3/28	0/3/14
Maximum disease extent:		
Proctitis	7	*NA* ^1^
Colitis ^2^	26	
Disease duration, mean months (range)	118 (3–420) ^3^	*NA*
Remission duration, mean months (range)	15 (1–61) ^3^	*NA*
Mayo endoscopic sub score, 0/1	24/9	*NA*
Medication:		
No treatment	8	3
5-ASA	17	0
Anti-TNFα	6	0
Immunosuppressants	7	0
Corticosteroid	1	0
α4β7 integrin inhibitor	1	0
Vitamin/iron supplements	2	3
Proton-pump inhibitor	0	1
Thiazides	0	2
Statins	0	2
Inhaler (β2-agonist, steroid)	1	3
Thyroid hormone	0	2
Antihistamine	2	1
Contraception	3	2
Anti-epileptic	1	0

^1^ NA, not applicable. ^2^ Either left-sided, extensive colitis or pancolitis. ^3^ Data from 2 patients missing.

**Table 2:** Basic SCC and responses to in vitro pharmacological interventions.

Baseline	UC (µA · cm^−2^)		Controls (µA · cm^−2^)		*p* value	
	107.6 ± 8.6 ^a^		107.5 ± 16.7 ^a^		0.99	
ENaC inhibition	−35.1 ± 7.5 ^b^		−37.2 ± 8.4 ^b^		0.85	
Baseline after ENaC inhibition	70.8 ± 7.5 ^a^		66.0 ± 14.4 ^a^		0.77	
Non-specific PDE inhibition	22.0 ± 3.7 ^b^		23.8 ± 5.2 ^b^		0.77	
COX-1 inhibition	−31.3 ± 3.8 ^b^		−26.2 ± 4.7 ^b^		0.40	
COX-2 inhibition	−51.6 ± 10.5 ^b^		−21.0 ± 3.7 ^b^		***0.01***	
**PGE_2_ concentration response**	**EC_50_ (nM)**	**R_max_ (µA cm^−2^)**	**EC_50_ (nM)**	**R_max_ (µA cm^−2^)**	**EC_50_ (nM)**	**R_max_**
High-affinity receptor	14.2 ± 2.7	60.9 ± 5.8	11.0 ± 2.4	52.1 ± 6.9	0.39	0.34
Low-affinity receptor	588.8 ± 106.7	83.1 ± 11.2	493.2 ± 110.6	84.7 ± 13.4	0.54	0.93

First row shows baseline short-circuit current (SCC) measured 10 min after mounting (UC: n = 33, controls: n = 15). Second row: inhibition of ENaC mediated sodium absorption induces a decrease in SCC (UC: n = 32, controls: n = 15). Third row: after ENaC inhibition, the new baseline SCC is driven by anion secretion. Fourth row: SCC increase after non-specific PDE inhibition (UC: n = 31, controls: n = 11). Rows five and six show SCC response (decrease) to COX-1 (UC: n = 27, controls: n = 12) and COX-2 inhibition (UC: n = 28, controls: n = 11), respectively. Rows seven and eight include half maximal effective concentration (EC_50_) and maximal receptor response (R_max_) of a high- and a low-affinity PGE_2_ receptor (UC: n = 19, controls: n = 12). SCC is recorded as µA · cm^−2^. n = number of patients included in experiment. Due to unstable and/or non-vital biopsies after mounting in MUAS chambers, not all patients are represented in this section. ^a^ Data are shown as mean SCC ± SEM. ^b^ Data are shown as mean ∆SCC ± SEM.

## Abbreviations

cMH	Complete mucosal healing
COX	Cyclooxygenase
DRA	Downregulated-in-adenoma
EC_50_	Half maximal effective concentration
ENaC	Epithelial sodium channel
eMH	Endoscopic mucosal healing
GI	Gastro-intestinal
hMH	Histological mucosal healing
IBD	Inflammatory bowel disease
MH	Mucosal healing
MUAS	Mini-Ussing-air-suction system
NSAID	Non-steroid anti-inflammatory drug
PDE	Phosphodiesterase
PGE_2_	Prostaglandin E2
qRT-PCR	Quantitative reverse transcription polymerase chain reaction
R_max_	Maximum response
SCC	Short-circuit current
SEM	Standard error of the mean
SD	Standard deviation
TJ	Tight junction
UC	Ulcerative colitis
WB	Western blot
